# MeURep: A novel user reputation calculation approach in personalized cloud services

**DOI:** 10.1371/journal.pone.0217933

**Published:** 2019-06-21

**Authors:** Jianlong Xu, Xin Du, Weihong Cai, Changsheng Zhu, Yindong Chen

**Affiliations:** 1 Department of Computer Science, Shantou University, Shantou, China; 2 Key Laboratory of Intelligent Manufacturing Technology (Shantou University), Ministry of Education, Shantou, China; 3 Guangdong Key Laboratory of Big Data Analysis and Processing, Guangzhou, China; RMIT University, AUSTRALIA

## Abstract

User reliability is notably crucial for personalized cloud services. In cloud computing environments, large amounts of cloud services are provided for users. With the exponential increase in number of cloud services, it is difficult for users to select the appropriate services from equivalent or similar candidate services. The quality-of-service (QoS) has become an important criterion for selection, and the users can conduct personalized selection according to the observed QoS data of other users; however, it is difficult to ensure that the users are reliable. Actually, unreliable users may provide unreliable QoS data and have negative effects on the personalized cloud service selection. Therefore, how to determine reliable QoS data for personalized cloud service selection remains a significant problem. To measure the reliability for each user, we present a cloud service selection framework based on user reputation and propose a new user reputation calculation approach, which is named MeURep and includes L1-MeURep and L2-MeURep. Experiments are conducted, and the results confirm that MeURep has higher efficiency than previously proposed approaches.

## Introduction

In the age of Internet of Things (IoT), cloud services have been the widespread concern in many realms [1–3]. In cloud environments, large amounts of services are provided for users, such as the computing power, storage, platforms, software, data storage service, and data access service, *etc*. [4–6]. Specifically, based on service-oriented architecture (SOA), currently, cloud services have become the underlying components in building high-quality cloud computing applications [7], [8].

With the exponential increase in number of cloud services, many equivalent or similar candidate services are provided for users, which causes great difficulty in selecting the proper services that provide the best performance for each user. Therefore, it is necessary to explore efficient techniques of personalized service selection. To select the optimal services from multitudinous services, the quality of service (QoS) is generally used as an important criterion [9], [10]. As a nonfunctional requirement, QoS is an important selection criterion to select the candidate cloud services [11], [12]. The QoS properties include the response time, invocation failure rate, etc. Commonly, different users observe different QoS properties when they invoke the same cloud service, which is named personalized QoS [13], [14]. In addition, similar users have similar QoS data when they invoke the same services. Based on these QoS data, a user can select an optimal service if this user knows in advance the QoS data of the services provided by other users [15]. For example, users U1 and U2 are located in the same city. We suppose that U1 had invoked services S1 and S2, which have similar functions, and the response time of invoking S1 is longer than the time of invoking S2; thus, S2 is optimal. When U2 wants to select services S1 or S2, U2 will give priority to S1. However, if U1 is an unreliable user, U2 will make a wrong choice. Under the circumstances, when making service selections, it is unreasonable to assume that all users are reliable. Because of the complexity of real networks, many users on the network provide unreliable QoS data. For example, if U1 and U2 are competitors, then each of them may provide malicious data for each other. Under this circumstance, the users may be simultaneously service providers; then, they may provide good QoS data for themselves and bad QoS data for their competitors. In other cases, some users are pranksters and may provide false data (random, maximal or minimal values) instead of real data. Therefore, unreliable users are very detrimental to service selection. Malicious information provided by unreliable users may disrupt the service choices of other users. On this account, we consider the users’ reputation.

Generally, reputation concerns the global opinions from the specific social community for a specific target, and it reflects the capability and will of the target to fulfill its promise [16]. Regarding personalized service selection, a higher reputation of a user corresponds to more accurate service selection performance, and vice versa. The users with high reputation will provide reliable QoS data, which generate more reliable conditions for other users to invoke suitable services. On the contrary, the users with low reputation hinder further service invocations because of the relatively high risk of service invocations. Therefore, accurate reputation values will facilitate users to make a suitable decision and promote the development of the cloud service. However, with the variability and uncertainty of user behavior, it is meaningless that the users directly submit their reputation. It is necessary to explore a reasonable method to obtain the user reputation.

As mentioned, unreliable users strongly affect the cloud services selection. According to the unreliable QoS data, users may select unsuitable or bad services. To address this issue, it is necessary to evaluate the reliability of users in the cloud services environment. In this paper, we present a user reputation calculation model, which is named MeURep. In our approach, the reputation calculation model is based on the historical QoS data submitted by other users. Our model assumes that each user has invoked the services and observed the QoS data. The user reputation is calculated based on the difference among the QoS data of other users. Iteratively, MeURep computes the user reputation until it converges to fixed values. Based on MeURep, we develop two algorithms with the following advantages:

1. Robustness. The algorithms are robust to unreliable QoS data.
2. Parallelization. The calculation of the user reputation of each user is independent and parallel computation, which can be easily implemented.
3. Efficiency. Our algorithms do not need to adjust the parameters in practice and can more quickly reach the convergence values than other algorithms.

The main contributions of this paper are summarized as follows:

1. We presented a new reliable framework of the reputation model for cloud services.
2. The reputation calculation model named MeURep is represented to calculate the reputation of each user based on the historical QoS data provided by the users in personalized cloud services.
3. Theoretical and experimental analyses show that our approach is more simple and effective.

The remainder of this paper is organized as follows. In Section 2, we review the related work. Then, our reputation model is proposed in Section 3. In Section 4, we conduct the experiments and show the results. The conclusion and future work are summarized in Section 5.

## Background and related work

In this section, we review the background and related work from three aspects: cloud services, personalized QoS of cloud services and reputation calculation approaches.

### Cloud services

In recent years, cloud services have become increasingly popular; tens of thousands of cloud services are emerging on the Internet. Generally, cloud services can be classified into three service models according to the needs of IT users [17], [18]:

1. SaaS (Software as a Service): provides users with the provider’s applications, which are accessible from various client devices through either a thin client interface, such as a web browser or a program interface.
2. PaaS (Platform as a Service): provides a platform for users to deploy onto the cloud infrastructure consumer-created or acquired applications (*e.g*. programming, libraries, services, *etc*.).
3. IaaS (Infrastructure as a Service): provides an environment for the users to deploy, run and manage virtual machines and storage.

With the vigorous development of the cloud services, many identical or similar services are offered by IT companies. For storage services, there are many IT companies including Amazon Simple Storage Service, Google Cloud Storage, and Microsoft Azure Storage. For database services, there are Google BigTable, Amazon SimpleDB, Fathom DB, Microsoft SDS, *etc*. These services are offered online, and the number is growing [19]. With the expansion of services on the Internet, how to select a suitable service from a set of equivalent cloud providers for users becomes an important challenge.

### Personalized QoS of cloud services

Personalized QoS is an important research topic in cloud computing and service computing. Except for functional QoS requirements (*e.g*., computation, database, storage, document management, *etc*.), nonfunctional of QoS (*e.g*., response time, throughput, *etc*.) are also extensively studied in recent years [20], [21]. Many QoS-based approaches have been proposed for cloud service composition, cloud service selection, etc. Pan *et al*. [22] proposed a trust-enhanced cloud service selection model based on QoS analysis; they used the trust-enhanced similarity to find similar trusted neighbors and predict the missing QoS data as the basis of the cloud service selection and recommendation. Wu *et al*. [23] focused on selecting skyline services in the dynamic environment for the QoS-based service composition; they proposed a skyline service model and a novel skyline algorithm to maintain dynamic skyline services. Zheng *et al*. [10] aimed to assist cloud uses to identify services; they proposed a collaborative filtering approach using the Spearman coefficient to recommend cloud services.

However, many previous studies did not consider the reliability of personalized QoS. Thus, to make cloud services selection more reasonable, we propose a QoS-based user reputation calculation approach.

### Reputation calculation approaches

The reputation calculation approach has been widely concerned by many scholars. Generally, reputation calculation approaches can be divided into two types: content-driven and user-driven. The principle of the content-driven approach is that the users’ reputation is calculated according to the quality and quantity of the user-generated content and the survival time of these contents. The principle of the user-driven approach is that the system makes a credit or reliability analysis according to the rating of the user feedback. Clearly, the user reputation calculation for cloud services is the user-driven type. In the current related research in the area of service computing, most works focused on the service side’s reputation and studied how to avoid the adverse effect from the feedback data of unreliable users. According to the feedback data from unreliable users, [24] introduced a reputation measurement approach based on the user similarity and cumulative sum to test the unreliable user feedback. Li *et al*. [25] also considered the effect of the similarity among users and proposed the peer trust model to evaluate the reliability of users. Su *et al*. [21] studied the trust perception approach for service recommendation and used QoS values to calculate the user reputation based on clustering algorithms. Wang *et al*. [26] introduced the feedback verification, validation, and feedback test to evaluate the service reputation. They calculated the users’ reputation through a statistical average approach using the QoS values of user feedback. In order to minimize the number of malicious services, Abdel Wahab et.al [27, 28] proposed a trust framework that allows services to establish credible trust relationships. Li et.al [29] presented a trust assessment framework for the security and reputation of cloud services, in this framework, they present a reputation-based trust assessment method, which is based on feedback rating derived from the cloud service providers. From the protocol perspective, Dou et.al [30] presented a distributed trust evaluation protocol for intercloud. From different perspectives and viewpoints, these approaches can be effective for service reputation.

Unlike the perspective of service, in this paper, we mainly focus on the perspective of the users. Our approach is based on the users’ QoS data and can be applied to personalized service selection, service composition, and service recommendation. In the related studies, Rong-Hua Li *et al*. [31] introduced six reputation calculations based on convergence algorithms. Baichuan Li *et al*. [32] proposed a topic-biased model (TBM) to estimate the user reputation applied in rating systems. In our preliminary study [33], we use the L1-AVG algorithm to calculate the users’ reputation. However, these approaches are also affected by the parameter settings; thus, there is still room for improvement in terms of effectiveness. Based on the former researchers, we attempt to explore a more effective and direct approach to obtain the users’ reputation.

## Approach

This section describes the approach and algorithms for user reputation. First, we present the notations and definitions. Then, we present a system framework and propose two algorithms. Finally, we analyze the time complexity for our MeURep algorithms.

### Notations and definitions

Let there be *m* different users *U* = {*u*_1_, *u*_2_,⋯, *u*_*m*_} and *n* services *S* = {*s*_1_, *s*_2_,⋯, *s*_*n*_}. In this case, service invocations will produce a user-service QoS matrix with respect to each QoS property. We can represent the user-service matrix as an *m*×*n* matrix *Q* ∈ *R*^*m*×*n*^. In this matrix, each entry *q*_*ij*_ (*i* ≤ *m*, *j* ≤ *n*) denotes the QoS property, which indicates that if the *i*^*th*^ user invokes the *j*^*th*^ cloud service, it will generate a QoS value. In this matrix, each row and column denotes a service user and a candidate service, respectively, and each entry in the matrix denotes the QoS data observed by a user when invoking a service. If the *i*^*th*^ user did not invoke the *j*^*th*^ cloud service before, then *q*_*ij*_ = null. The reputation of users can be represented as *R* = {*r*_1_, *r*_2_,⋯, *r*_*m*_}. We assume that the users’ reputation ranges from 0 to 1 (0 ≤ *r*_*i*_ ≤ 1). The most unreliable user’s reputation is 0, whereas the most reliable user’s reputation value is 1. Our goal of the reputation calculation is to excavate the information from the QoS property values of each user.

### System framework

**Fig 1:**
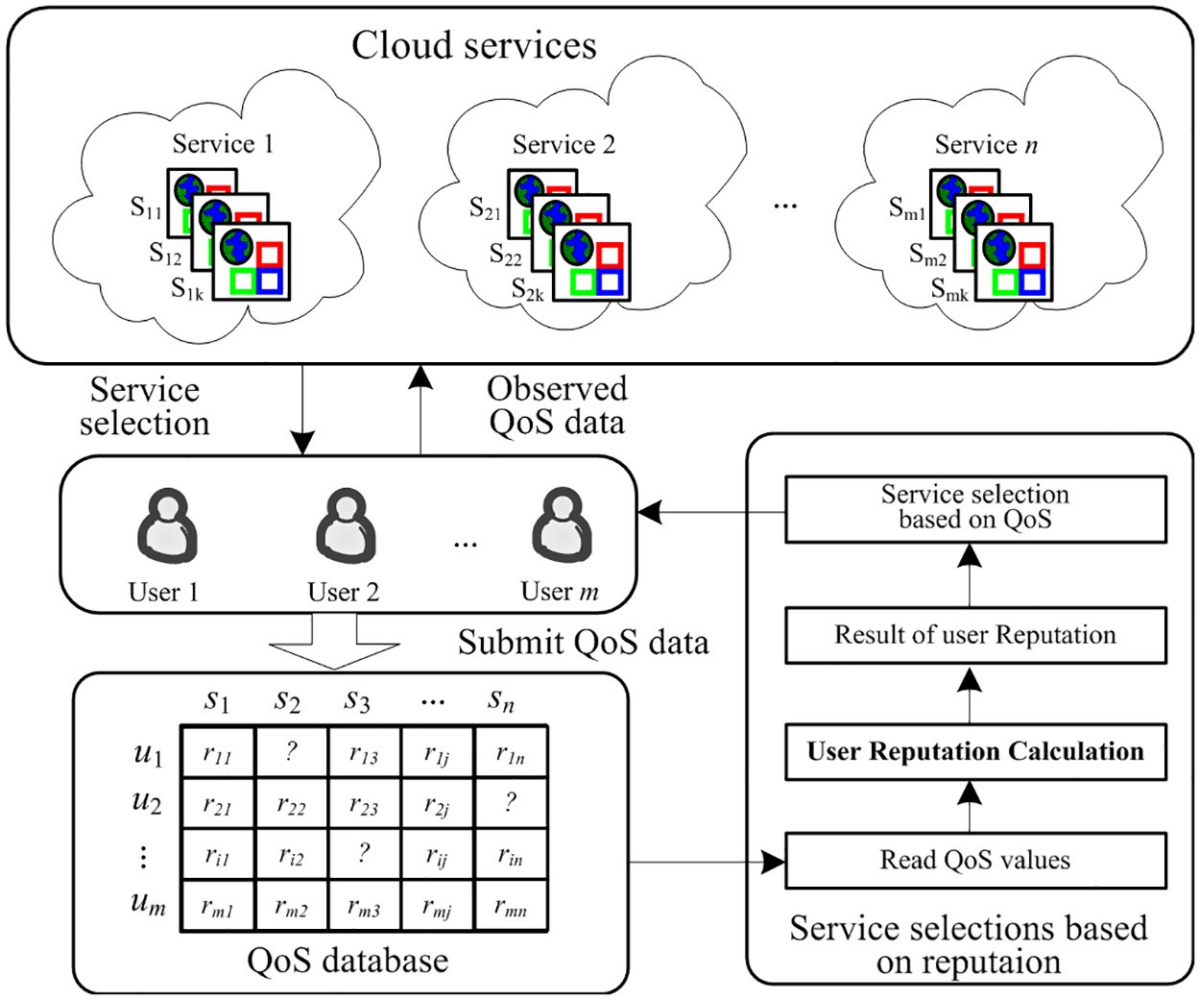
Cloud service selection based on user reputation.

We present a framework for cloud service selection based on user reputation in Fig 1. In this framework, the reputation calculation plays an important role. As Fig 1 shows, there are many types of cloud services on the Internet, each of which has many similar or equal services. The users invoke the cloud services and submit their observed QoS data to the QoS database, and the cloud service selection module performs the service selection after calculating the user reputation. Noteworthy, the QoS data can be measured at the server side or the user side. In this framework, QoS data are provided by the user side, which is personalized. In contrary to rating values in rating systems, the QoS data fluctuate in an uncertain range. Therefore, the reputation calculation models designed for the rating system may not be suitable for cloud services.

**Fig 2:**
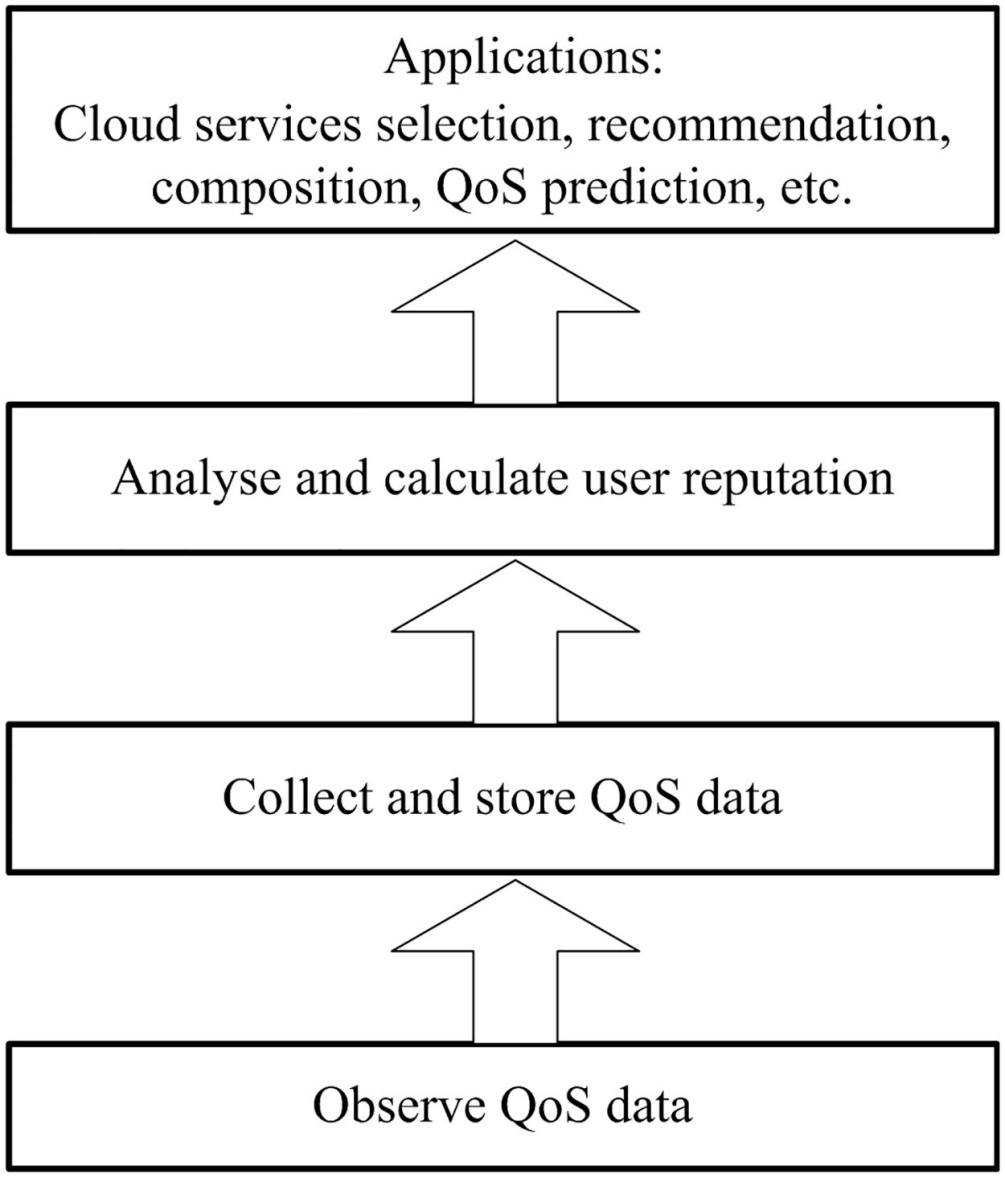
User reputation and various applications.

The user reputation can also be applied in cloud service recommendation, prediction, *etc*. As Fig 2 shows, the entire process of the user reputation applications contains four parts: observe QoS data, collect and store the QoS data, analyze and calculate the user reputation, and applications. The first three parts can be accomplished in real time. This paper mainly focuses on the user reputation calculation.

### User reputation calculation model

To compare with our approach, first, we will introduce a reputation calculation algorithm, which was proposed in [31], named L1-AVG. This algorithm can be expressed as:

$$\begin{array}{c} \left\{ \begin{array}{c} A_{j}^{k+1}=\frac{1}{|H_{j}|}\sum_{u_{i}\in H_{j}}q_{ij}r_{i}^{k}\\ r_{i}^{k+1}=1-\frac{d}{|O_{i}|}\sum_{s_{j}\in O_{i}}|q_{ij}-A_{j}^{k+1}| \end{array}\right. \end{array}$$ (1)

In (1), *q*_*ij*_ denotes a certain QoS value, *k* is the *k*^*th*^ iteration, $r_{i}^{k}$ is the reputation *r*_*i*_ in the *k*^*th*^ iteration, and *A*_*j*_ is the average QoS value for the *j*^*th*^ service. After *k*+1 iterations, *A*_*j*_ is changed to $A_{j}^{k+1}$. When the *j*^*th*^ service is invoked, it will be recorded and represented as *H*_*j*_. |*H*_*j*_| is the number of users who have invoked the *j*^*th*^ service. Similarly, when the *i*^*th*^ user invokes some services, it will be recorded and represented as *O*_*i*_. |*O*_*i*_| is the number of services that have been invoked by the *i*^*th*^ user. To ensure that *r*_*i*_ ranges from 0 to 1, damping coefficient *d* plays a part in the regulation of the calculation result. For better results, the L1-AVG have to adjust its the parameter *d* according to different data. Such as in our experiments, *d* is set to 0.02 for the response time datasets while *d* is set to 0.01 for the throughput datasets. This shows that it is very inconvenient.

From (1), the reputation value is obtained based on the degree of deviation in each convergence process. However, it also has its scope of application. It is applicable to situations where the value is within a certain range. Actually, QoS data are highly skewed with large variances. The unreliability user may supply unlimited values. If an unreliable user submits negative data, the average value may be negative, and the reputation calculation results may be negative, which is out of range of the defined reputation. Meanwhile, although the L1-AVG algorithm uses damping coefficients to adjust the calculation results in each convergence process, it is not convenient to determine the value of damping coefficients.

To address this problem, we propose a user reputation calculation approach based on the median value analysis, named MeURep. MeURep includes two algorithms: L1-MeURep and L2-MeURep.

The L1-MeURep algorithm is represented as:

$$\begin{array}{c} \left\{ \begin{array}{c} T_{j}^{k+1}=\underset{u_{i}\in H_{j}}{med}\left(q_{ij}r_{i}^{k}\right)\\ r_{i}^{k+1}=1-\frac{1}{|O_{i}|}\sum_{s_{j}\in O_{i}}(\frac{|q_{ij}-T_{j}^{k+1}|}{\underset{s_{j}\in O_{i}}{max}\left|q_{ij}-T_{j}^{k+1}\right|}) \end{array}\right. \end{array}$$ (2)

In (2), *T*_*j*_ is the median QoS value for the *j*^*th*^ service invoked by the users, and $T_{j}^{k+1}$ is *T*_*j*_ after the *k* + 1^*th*^ iteration. The meanings of $r_{i}^{k}$, *H*_*j*_, *O*_*i*_, |*O*_*i*_| are identical to those in (1). Specifically, in the worst case, the median may be negative when half of the users’ data are negative. the median also comes from an unreliable user when more than half the users are unreliable. In this case, the system has become meaningless. Therefore, our method is suitable for the situation that the percentage of unreliable users is less than half.

Like L1-AVG, the calculation process of L1-MeURep is based on the convergence. Unlike L1-AVG, we use the median value instead of the average value and calculate the maximum of $\left|q_{ij}-T_{j}^{k+1}\right|$. *r*_*i*_ is largely determined by *q*_*ij*_ and $T_{j}^{k+1}$. The main idea of L1-MeURep can be simply represented that if the QoS data provided by a certain user is very different from the median, then this user is probably not reliable. However, in extreme cases, if the quantity of the unreliable users is more than the number of reliable users, the median value will come from an unreliable user, and the QoS data provided by a reliable user may be very different from the median. The methodology of L1-MeURep is in Algorithm 1.

In Algorithm 1, we first initialize the parameters. In the initialization, *k* = 0 and $r_{i}^{0}=1$. Then, the median QoS value for the *j*^*th*^ service and the reputation of the *i*^*th*^ user are calculated according to (2) using the iterative approach. When *k* is more than RMaxI (the maximum number of iterations) or the absolute of $r_{i}^{k+1}-r_{i}^{k}$ satisfies the required accuracy (less than thresholds), the algorithm will be terminated and outputs the user reputation.

In (2), one of the key steps is to calculate the absolute of $q_{ij}-T_{j}^{k+1}$, and we also try another computation mode as follows.

**Algorithm 1** L1-MeURep algorithm

**Require**: matrix *Q*; *H*_*j*_ = 0; *O*_*i*_, RMaxI; threshold;

**Ensure**: user’s reputation *r*_*i*_;

1: initialize *k* = 0; $r_{i}^{0}=1$; |*O*_*i*_| = 0;

2: **while** *k*¡RMaxI **do**

3:   **for**(*j* = 1; *j* <= *n*; *j*++)

4:    **for**(*i* = 1; *i* <= *length*(*H*_*j*_); *i*++)

5:    Med(*j*) = median(Q[1: |*H*_*j*_|, *j*])

6:    **end for**

7:    $T_{j}^{k+1}\leftarrow Med\left(j\right)\times r_{i}^{k}$

8:   **end for**

9:    **for**(*i* = 1; *i* <= *m*; *i*++)

10:    SumR = 0; CountR = 0;

11:    **for**(*j* = 1; *i* <= *length*(*O*_*i*_); *j*++)

12:     **if** *q*_*ij*_ > 0

13:  SumR = SumR+absolute($q_{ij}-T_{j}^{k+1}$)/max(absolute($q_{ij}-T_{j}^{k+1}$));

14:    |*O*_*i*_| = CountR++;

15:     **end if**

16:    **end for**

17:     $r_{i}^{k+1}$ ← 1-SumR/|*O*_*i*_|;

18:   **end for**

19:  **if** absolute($r_{i}^{k+1}-r_{i}^{k}$) ¡ threshold

20:  **then** break;

21: **end while**

The L2-MeURep algorithm is represented as:

$$\begin{array}{c} \left\{ \begin{array}{c} T_{j}^{k+1}=\underset{u_{i}\in H_{j}}{med}\left(q_{ij}^{}r_{i}^{k}\right)\\ r_{i}^{k+1}=1-\frac{1}{|O_{i}|}\sum_{s_{j}\in O_{i}}(\frac{{\left(q_{ij}^{}-T_{j}^{k+1}\right)}^{2}}{\underset{s_{j}\in O_{i}}{max}{\left(q_{ij}^{}-T_{j}^{k+1}\right)}^{2}}) \end{array}\right. \end{array}$$ (3)

Unlike L1-MeURep, we change the absolute mode to the square mode for $q_{ij}-T_{j}^{k+1}$ in (3). Since the pseudo code of L2-MeURep algorithm is similar to the L1-MeURep algorithm, we omit the details which like in Algorithm 1 for it. From (2) and (3), there is no damping coefficient; thus, it is more convenient than L1-AVG algorithm.

The complexity analysis of L1-MeURep and L2-MeURep is as follows. We assume that the amortized cost in a single iteration is *C*(|*G*|), where |*G*| is the total number of edges in the bipartite graph. As a result, for *k* iterations, the total running time of MeURep algorithms is *C*(*k*|*G*|).

## Experiment

In this section, we conduct experiments to validate our MeURep approach. Our experiments are intended to verify the rationality of our proposed theorems and compare our approach with the other approach.

### Experimental setup

The purposes of the experiments are to use the data to calculate each user’s reputation value and verify the validity of our algorithms. In our experiments, we use the real-world reliable users’ datasets released by Zheng *et al*. [34]. From these datasets, we use two matrices. Each of them is a 339×5825 matrix, i.e., 339 users and 5825 services. In these two matrices, the entries are the vectors of QoS values observed by a service user on a Web service, which are response time properties and throughputs, respectively. In the experiment, in order to make the experiments more realistic, we mixed many unreliable users which are generated at random into these 339 users. Furthermore, the number of added unreliable users may also impact the algorithm’s performance; thus, we adjusted the proportion of unreliable users in the datasets to different levels: 2%, 4%, 6%, 8%, and 10%. Table 1 brief describe the 379×5825 throughput matrix, which contains approximately 10% of unreliable users. In our datasets, the range of the response time is 0∼20*s*, and the range of the throughput is 0∼7000*kbps*. Due to page limitations, we don’t describe the response time matrices and throughput matrices of the other different proportions. In this way, we believe the conduct experiments are better and more persuasive. It is worth noting that our matrices are the off-line data of the response time properties and throughputs, so their density is not sparse relative to real-time data. If the data is missing at a certain position in the matrix, we randomly assign it to a non-negative number.

**Table 1:** 379×5825 user-service throughput matrix.

	Service 1	Service 2	Service 3	Service 4	Service 5	Service 6	…	Service 5825
**User 1**	0.9380	15.2670	21.9780	7.9680	7.8740	7.0250	…	4.3660
**User 2**	2.3410	10.9280	15.9570	5.6020	5.5860	26.0860	…	2.48900
**User 3**	2.8860	17.6990	25.7510	9.0900	9.1320	8.7200	…	11.32000
**…**	…	…	…	…	…	…	…	…
**User 378**	45.4312	49.187	49.673	50.511	49.735	49.411	…	50.583
**User 379**	50.6160	50.4732	50.3516	50.8308	50.2147	50.9259	…	50.7567

According to the range of reputation values defined in Section 3, we further define the calculation average error of the reputation value as follows:

$$\begin{array}{c} E_{re}=\frac{\sum_{i=1}^{N_{re}}\left(1-r_{i}\right)}{N_{re}} \end{array}$$ (4)

$$\begin{array}{c} E_{ur}=\frac{\sum_{i=1}^{N_{ur}}\left(r_{i}-0\right)}{N_{ur}} \end{array}$$ (5)

In (4) and (5), *E*_*re*_ and *E*_*ur*_ are the average error of the reputation values for reliable users and unreliable users, respectively. *N*_*re*_ and *N*_*ur*_ are the numbers of reliable users and unreliable users, respectively.

As we mentioned before, RMaxI is the maximum number of iterations, which aim is to avoid getting caught in endless iterations when the algorithm does not converge. In the following experiments, refer to [31] and our results, we set RMaxI as 10 and the threshold as 0.001. To better reflect the experimental results in the paper, we show in the figures is five randomly selected users from the 379×5825 matrices, whose number 1-4 are reliable, and number 5 is unreliable.

### Experimental results and discussion

We present the performance of different approaches in calculating the user reputation in this section. Specifically, we construct an experiment not only in different approaches but also in diverse datasets, which contain the varying proportion of unreliable users. The experimental results reflect the superiority of our methods in accuracy and efficiency from users reputation value and iteration processes.

**Fig 3:**
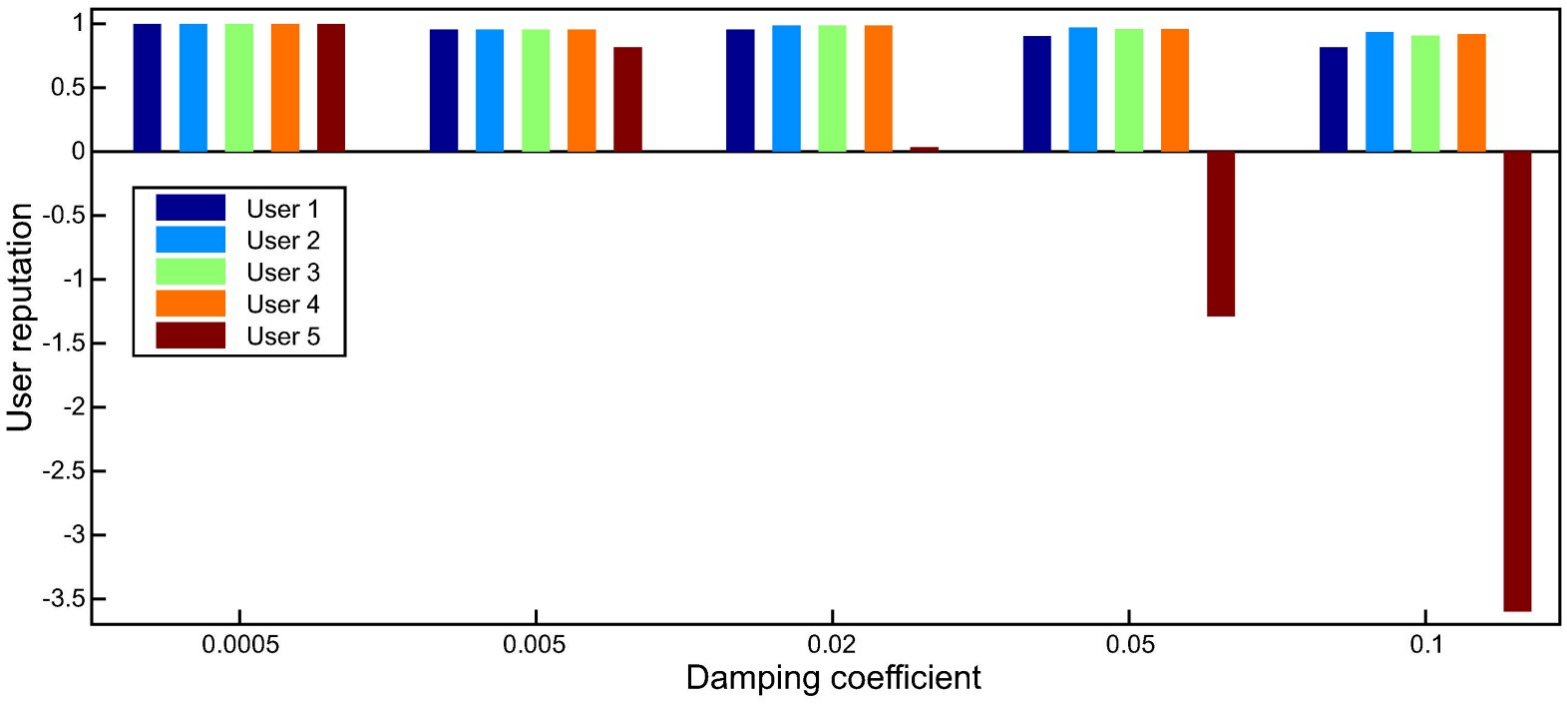
Different damping coefficients for L1-AVG (for response time).

For experiments using L1-AVG, we vary the damping coefficient *d* with different values to optimize it accordingly to achieve their optimal accuracy. Fig 3 shows the results of the users’ reputation values for different damping coefficients for L1-AVG.

Fig 3 shows that the value of user reputation significantly varies with different damping coefficients. For example, when *d* = 0.02, the reputation values of users 1-5 are 0.9644, 0.9836, 0.9834, 0.9804, and 0.0233, respectively. The average error *E*_*re*_ is 0.0220, and *E*_*ur*_ is 0.0233. In Fig 3, we can find the reputation value of user 5 looks identical to those of users 1-4 when *d* = 0.0005. Since user 5 is unreliable, *d* = 0.0005 is unreasonable. When *d* = 0.05 or 0.1, the reputation value of user 5 is negative, which is out of the defined range of reputation, and it is also unreasonable. The reason can be explained as follows: the value of $d*\sum |q_{ij}-A_{j}^{k+1}|$ is too big, and if we divide it by |*O*_*i*_|, the result may be in excess of 1. Therefore, to obtain satisfactory results, the damping coefficient is adjusted for many times. By this way, we conclude that the optimal value for response time is 0.02 and the optimal value for throughput is 0.001.

**Fig 4:**
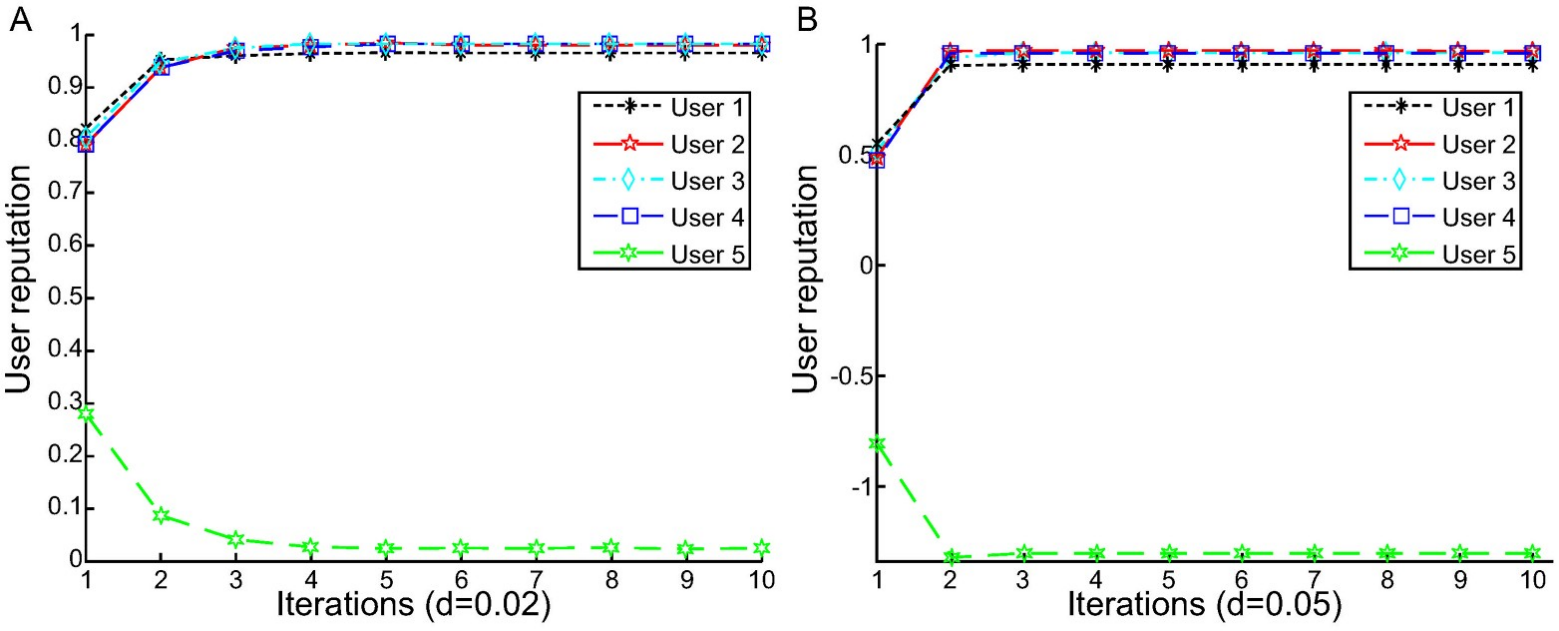
User reputation calculation iteration process based on L1-AVG (for response time). A: User reputation calculation iteration process based on L1-AVG(d = 0.02). B: User reputation calculation iteration process based on L1-AVG(d = 0.05).

Fig 4 illustrates the iteration process of L1-AVG. We can see the following:

1. As a whole, the iteration processes of users 1-4 are similar. They are first in an unstable state and subsequently converge to a fixed value after a few iterations.
2. When d = 0.02 (Fig 4(a)), L1-AVG achieves convergence after three iterations for user 1. The number of iterations for users 2-5 is four. For reliable users 1-4, the reputation value curve rises until it reaches a stable value. For user 5, the reputation curve is in a descending state until it reaches a stable value. In the iteration process, the iterative initial values of users 1-4 is more than that of user 5 in the first step of iterations.
3. When d = 0.05 (Fig 4(b)), the iterations is three for users 1, 3 and 5, whereas the reputation values achieve convergence after three iterations for users 2 and 4.

Therefore, the number of iterations is not inconsonant. For different decay constants, the number of iterations to converge is different in the user reputation calculation iteration process. Even for the same decay constant, the number of iterations to converge also varies.

In the following content, we conduct experiments to validate our MeURep approach. We use a part of the response time and throughput dataset and verify our approaches L1-MeURep and L2-MeURep.

**Fig 5:**
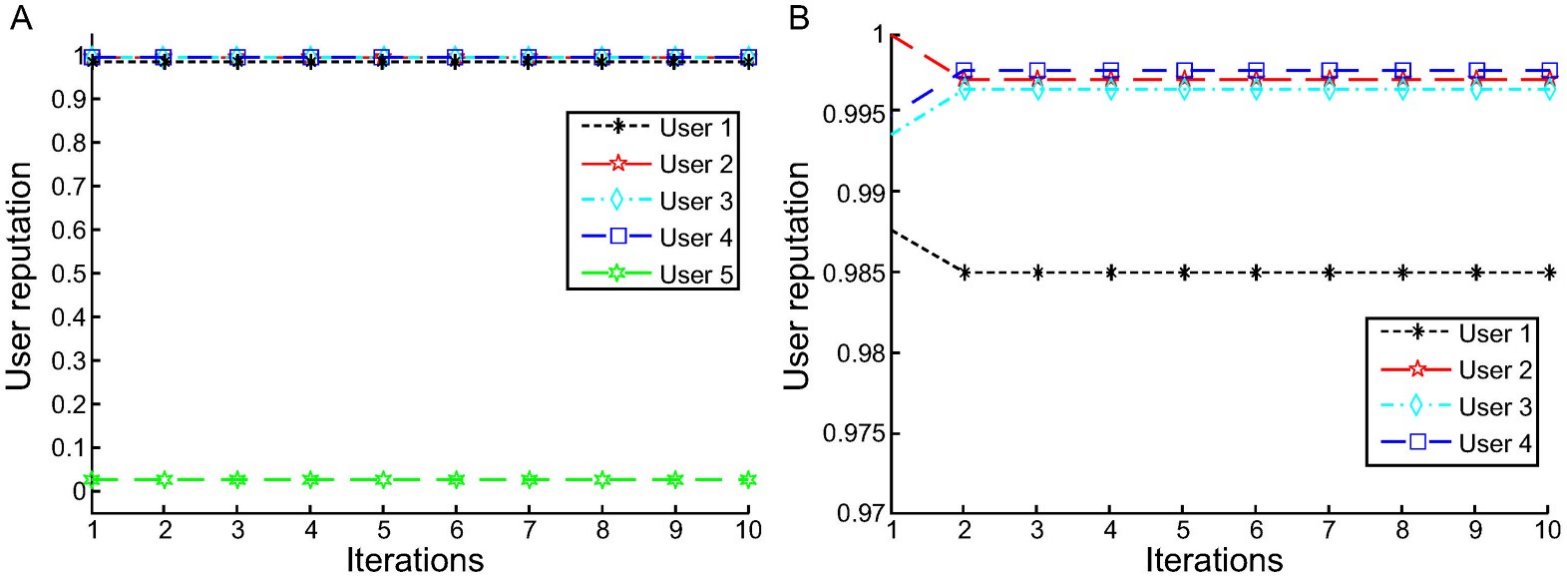
User reputation calculation iteration process based on L1-MeURep (for response time). A: User reputation calculation iteration process based on L1-MeURep for user 1 to 5. B: User reputation calculation iteration process based on L1-MeURep for user 1 to 4.

The experimental results of L1-MeURep for the response time are illustrated in Fig 5. In Fig 5, the reputation values of users 1-5 are 0.9850, 0.9972, 0.9966, 0.9978, and 0.0234, respectively. Fig 5(a) shows a bird’s eye view of the iteration process for users 1-5. Fig 5(b) indicates the details of users 1-4. We can observe the following:

1. L1-MeURep can quickly reach a convergence value after the second iteration.
2. The average error *E*_*re*_ is 0.00585; *E*_*ur*_ is 0.0234. Compared to L1-AVG (d = 0.02), the average error *E*_*re*_ declines by 73.4%; *E*_*ur*_ is almost identical.

**Fig 6:**
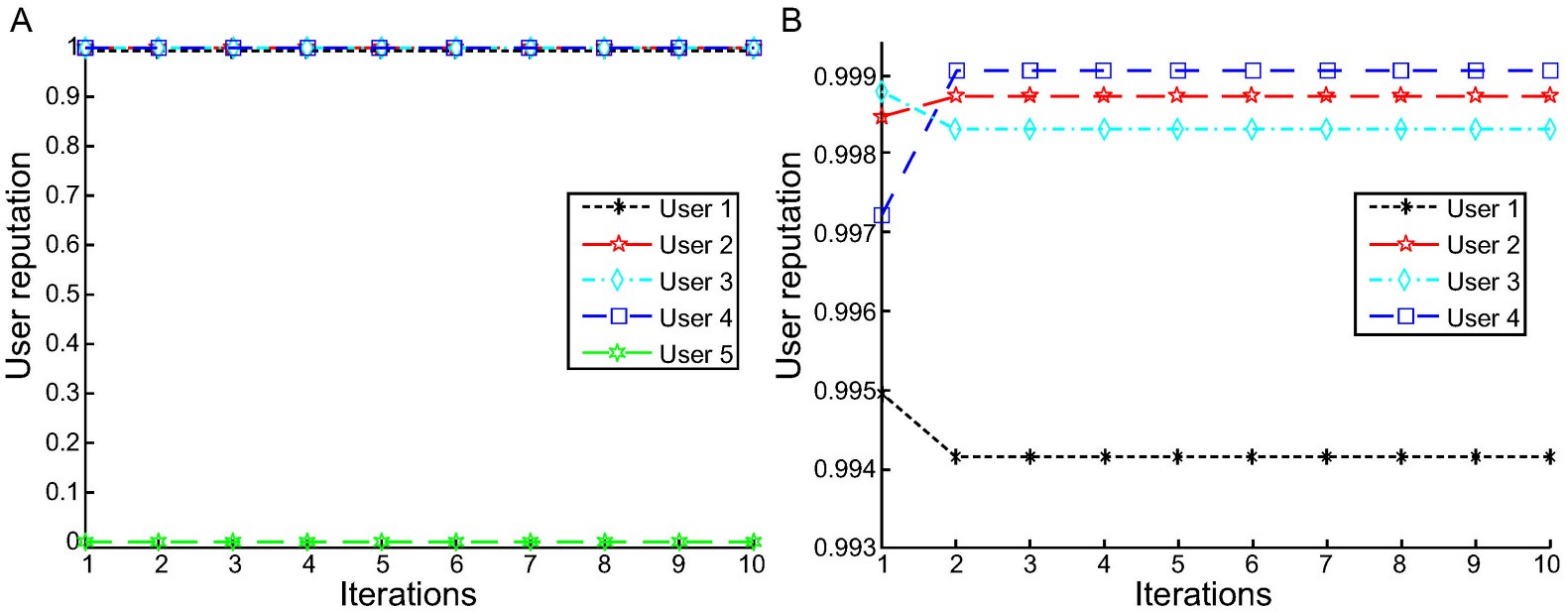
User reputation calculation iteration process based on L2-MeURep (for response time). A:The reputation values of user 1 to 5. B:The reputation values of user 1 to 4.

Fig 6 shows the experimental results of our approach L2-MeURep in terms of response time. The reputations of users 1-5 are 0.9942, 0.9987, 0.9983, 0.9991, and 0, respectively. It also quickly converges to a fixed value after two iterations. The average error *E*_*re*_ is 0.0024; *E*_*ur*_ is 0.

**Fig 7:**
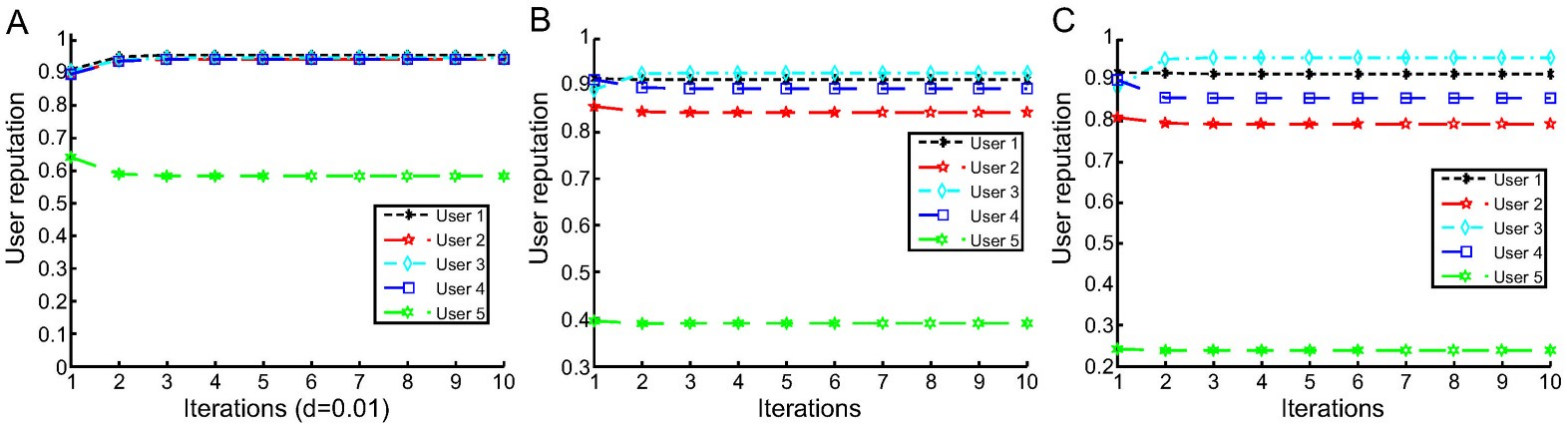
User reputation calculation iteration process based on different approaches (for throughput). A: L1-AVG B: L1-MeURep C: L2-MeURep.

The experimental results of using the throughput dataset including approximately 10% of unreliable users are illustrated in Table 2 and the iteration processes are shown in Fig 7. In addition, based on the reputation values of all users, we make the comparison of users reputation errors in different proportions(as shown in Table 3). We observe the following:

1. In all cases of the experiment, *E*_*re*_ and *E*_*ur*_ of L1-MeURep and L2-MeURep are less than of L1-AVG.
2. When we increase the proportion of unreliable users, *E*_*re*_ and *E*_*ur*_ are both increase under different approaches. However, compared L1-MeURep and L2-MeURep with L1-AVG, the growth rate of these metrics is obviously much lower.
3. Fig 7 denotes our MeURep approaches are also faster than L1-AVG when using the throughput dataset. In fact, the conclusion is still valid in other datasets.
4. Compared L1-MeURep with L2-MeURep, we observed that the *E*_*re*_ of L2-MeURep is less than L1-MeURep, but the *E*_*ur*_ is larger than it.

**Table 2:** User reputation calculation results. (For the proportion is 10%).

Qos	Method	User 1	User 2	User 3	User 4	User 5	User 6	User 7	…	User 378	User 379
RT	**L1-AVG(d = 0.02)**	0.9638	0.9739	0.9738	0.9719	0.9729	0.9719	0.9638	…	0.2905	0.2904
	**L1-MeURep**	0.9525	0.9928	0.9794	0.9939	0.9943	0.9928	0.9936	…	0.0191	0.0188
	**L2-MeURep**	0.9946	0.9995	0.9976	0.9995	0.9994	0.9996	0.9994	…	0.0371	0.0372
TP	**L1-AVG(d = 0.01)**	0.7637	0.7621	0.8060	0.8413	0.6748	0.7781	0.7194	…	0.5612	0.5613
	**L1-MeURep**	0.8983	0.8860	0.9264	0.9108	0.8890	0.8991	0.9051	…	0.2743	0.2745
	**L2-MeURep**	0.9712	0.9804	0.9745	0.9633	0.9577	0.9567	0.9718	…	0.3238	0.3239

**Table 3:** The comparison of user’s reputation errors in different proportions.

QoS	Method	percentage = 2%		percentage = 4%		percentage = 6%		percentage = 8%		percentage = 10%	
		*E*_*re*_	*E*_*ur*_	*E*_*re*_	*E*_*ur*_	*E*_*re*_	*E*_*ur*_	*E*_*re*_	*E*_*ur*_	*E*_*re*_	*E*_*ur*_
RT	L1-AVG(d = 0.02)	0.0123	0.2714	0.0155	0.2758	0.0192	0.2804	0.0230	0.2810	0.0277	0.2913
	L1-MeURep	0.0126	0.0149	0.0127	0.0171	0.0126	0.0181	0.0126	0.0186	0.0127	0.0191
	L2-MeURep	0.0013	0.0294	0.0013	0.0338	0.0013	0.0357	0.0012	0.0366	0.0013	0.0373
TP	L1-AVG(d = 0.01)	0.2536	0.5122	0.2553	0.5251	0.2577	0.5378	0.2603	0.5486	0.2638	0.5212
	L1-MeURep	0.1055	0.2712	0.1054	0.2721	0.1053	0.2729	0.1054	0.2736	0.1055	0.2744
	L2-MeURep	0.0370	0.3206	0.0367	0.3218	0.0367	0.3226	0.0365	0.3232	0.0364	0.3239

From the above experimental results, we find that our approach is more simple and efficient than the L1-AVG algorithm. First, it does not require a damping coefficient to adjust the calculation result. Therefore, it is unnecessary to tune parameters in the experiment. Second, for the L1-AVG algorithm, the average value *A*_*j*_ is strongly affected by unreliable user data (*e.g*., the data of user 379 in the Table 1 increase the average value, and the value $\left|q_{ij}-A_{j}^{k+1}\right|$ has a great change). By contrast, because of the quantity of the unreliable users accounts for a relatively small proportion in reality, our approach uses the median value to avoid being impacted by a specific abnormal data (*e.g*., regardless of how large or small the value of user 379 is, the median value does not change much). Since $\left|q_{ij}-T_{j}^{k+1}\right|$ is close to or equal to $max(\left|q_{ij}-T_{j}^{k+1}|\right)$, the reputation value of unreliable users is notably small. Third, our model is faster than L1-AVG. The reputations reach convergence values after two iterations in our algorithm (Fig 5(b)) but three iterations in L1-AVG (d = 0.02) (Fig 4(a)). Fourth, the experimental results show that our approach is more accurate than L1-AVG. In addition, for the response time experiments, the value *E*_*re*_ of L2-MeURep is better than L1-MeURep, but *E*_*ur*_ of L2-MeURep is worse than L1-MeURep. And for the throughput experiments, the result is the same. So L1-MeURep seems more suitable for identifying unreliable users. In a nutshell, the performance of L1-MeURep and L2-MeURep is difficult to distinguish between good or bad in our dataset, how to decide which one should be chosen when implementation reply on the actual situation.

## Conclusion and future work

In the cloud service environment, users usually need to select optimal services according to other users’ personalized QoS data to build various applications. However, in the complex network environment, some users may provide unreliable QoS data, which causes a negative effect on the service selection. Therefore, it is important to know the users’ reliability. To measure the users’ reliability, it is usually necessary to calculate the users’ reputation values. In this paper, we present a user reputation calculation approach, namely MeURep. First, we present a cloud service selection framework based on user reputation. Then, we propose MeURep algorithms called L1-MeURep and L2-MeURep. Finally, to verify the validity of our approach, we have conducted experiments on a real-world dataset. The experimental results show that our approaches have high efficiency compared to the other approach. Compared with L1-AVG, the average error *E*_*re*_ of our algorithm achieves 89.43% ∼ 95.31% improvement for response time and 85.41% ∼ 86.20% improvement for throughput at the different proportion of unreliable users. Similarly, the average error *E*_*ur*_ of our algorithm achieves 93.44% ∼ 94.50% improvement for response time and 47.05% ∼ 50.13% improvement for throughput.

In the future, to achieve better performance, we plan to take the subcategory information into consideration to improve the calculation quality. In addition, to improve the real-time ability, we will consider the online environment.

## Supporting information

S1 Dataset This dataset describes real-world QoS measurements, including both response time and throughput values. (RAR)

## References

[1] Bermbach D. Quality of cloud services: Expect the unexpected. IEEE Internet Computing. 2017;21(1):68–72. 10.1109/MIC.2017.1

[2] Mubeen S, Asadollah SA, Papadopoulos AV, Ashjaei M, Pei-Breivold H, Behnam M. Management of service level agreements for cloud services in IoT: a systematic mapping study. IEEE Access. 2018;6:30184–30207. 10.1109/ACCESS.2017.2744677

[3] Alkalbani AM, Gadhvi L, Patel B, Hussain FK, Ghamry AM, Hussain OK. Analysing cloud services reviews using opining mining. In: Advanced Information Networking and Applications (AINA), 2017 IEEE 31st International Conference on. IEEE; 2017. p. 1124–1129.

[4] Script GA. Soap Services,;. https://developers.google.com/apps-script/reference/soap/, 2017.

[5] Service ARD. SOAP API;. http://docs.aws.amazon.com/AmazonRDS/latest/UserGuide/using-soap-api.html, 2017.

[6] Service ASS. SOAP API;. http://docs.aws.amazon.com/AmazonS3/latest/API/APISoap.html, 2017.

[7] Koehler M, Benkner S. Design of an adaptive framework for utility-based optimization of scientific applications in the cloud. In: Proceedings of the 2012 IEEE/ACM Fifth International Conference on Utility and Cloud Computing. IEEE Computer Society; 2012. p. 303–308.

[8] Phaphoom N, Wang X, Samuel S, Helmer S, Abrahamsson P. A survey study on major technical barriers affecting the decision to adopt cloud services. Journal of Systems and Software. 2015;103:167–181. 10.1016/j.jss.2015.02.002

[9] ur Rehman Z, Hussain OK, Hussain FK, Chang E, Dillon T. User-side QoS forecasting and management of cloud services. World Wide Web. 2015;18(6):1677–1716. 10.1007/s11280-014-0319-8

[10] Zheng X, Da Xu L, Chai S. Qos recommendation in cloud services. IEEE Access. 2017;5:5171–5177. 10.1109/ACCESS.2017.2695657

[11] Zheng Z, Wu X, Zhang Y, Lyu MR, Wang J. QoS ranking prediction for cloud services. IEEE transactions on parallel and distributed systems. 2013;24(6):1213–1222. 10.1109/TPDS.2012.285

[12] Li W, Zhang P, Leung H, Ji S. A Novel QoS Prediction Approach for Cloud Services Using Bayesian Network Model. IEEE Access. 2018;6:1391–1406. 10.1109/ACCESS.2017.2779045

[13] Garg SK, Versteeg S, Buyya R. Smicloud: A framework for comparing and ranking cloud services. In: Utility and Cloud Computing (UCC), 2011 Fourth IEEE International Conference on. IEEE; 2011. p. 210–218.

[14] Serrano D, Bouchenak S, Kouki Y, Ledoux T, Lejeune J, Sopena J, et al. Towards qos-oriented sla guarantees for online cloud services. In: Cluster, Cloud and Grid Computing (CCGrid), 2013 13th IEEE/ACM International Symposium on. IEEE; 2013. p. 50–57.

[15] Wu H, Yue K, Hsu CH, Zhao Y, Zhang B, Zhang G. Deviation-based neighborhood model for context-aware QoS prediction of cloud and IoT services. Future Generation Computer Systems. 2017;76:550–560. 10.1016/j.future.2016.10.015

[16] Jiang D, Xue J, Xie W. A reputation model based on hierarchical bayesian estimation for web services. In: Computer Supported Cooperative Work in Design (CSCWD), 2012 IEEE 16th International Conference on. IEEE; 2012. p. 88–93.

[17] Cusumano M. Cloud computing and SaaS as new computing platforms. Communications of the ACM. 2010;53(4):27–29.

[18] Mell P, Grance T. The NIST definition of cloud computing. 2011.

[19] Luo X, Lv Y, Li R, Chen Y. Web service QoS prediction based on adaptive dynamic programming using fuzzy neural networks for cloud services. IEEE Access. 2015;3:2260–2269. 10.1109/ACCESS.2015.2498191

[20] Zeng L, Benatallah B, Ngu AH, Dumas M, Kalagnanam J, Chang H. QoS-aware middleware for web services composition. IEEE Transactions on software engineering. 2004;30(5):311–327. 10.1109/TSE.2004.11

[21] Sun L, Dong H, Hussain FK, Hussain OK, Chang E. Cloud service selection: State-of-the-art and future research directions. Journal of Network and Computer Applications. 2014;45:134–150. 10.1016/j.jnca.2014.07.019

[22] Pan Y, Ding S, Fan W, Li J, Yang S. Trust-enhanced cloud service selection model based on QoS analysis. PloS one. 2015;10(11):e0143448 10.1371/journal.pone.0143448 26606388 PMC4659544

[23] Wu J, Chen L, Liang T. Selecting dynamic skyline services for QoS-based service composition. Applied Mathematics & Information Sciences. 2014;8(5):2579 10.12785/amis/080557

[24] Shang-Guang W, Qi-Bo S, Fang-Chun Y. Reputation Evaluation Approach in Web Service Selection [J]. Journal of Software. 2012;6:003.

[25] Xiong L, Liu L. Peertrust: Supporting reputation-based trust for peer-to-peer electronic communities. IEEE transactions on Knowledge and Data Engineering. 2004;16(7):843–857. 10.1109/TKDE.2004.1318566

[26] Wang S, Zheng Z, Wu Z, Lyu MR, Yang F. Reputation measurement and malicious feedback rating prevention in web service recommendation systems. IEEE Transactions on Services Computing. 2015;8(5):755–767. 10.1109/TSC.2014.2320262

[27] Wahab OA, Bentahar J, Otrok H, Mourad A. A survey on trust and reputation models for Web services. decision support systems. 2015;74:121–134. 10.1016/j.dss.2015.04.009

[28] Wahab OA, Bentahar J, Otrok H, Mourad A. Towards Trustworthy Multi-Cloud Services Communities: A Trust-Based Hedonic Coalitional Game. IEEE Transactions on Services Computing. 2018;11(1):184–201. 10.1109/TSC.2016.2549019

[29] Li X, Wang Q, Lan X, Chen X, Zhang N, Chen D. Enhancing Cloud-Based IoT Security through Trustworthy Cloud Service: An Integration of Security and Reputation Approach. IEEE Access. 2019; p. 1–1.

[30] Dou Y, Chan H, Au MH. A Distributed Trust Evaluation Protocol with Privacy Protection for Intercloud. IEEE Transactions on Parallel and Distributed Systems. 2018; p. 1–1.

[31] Li RH, Xu Yu J, Huang X, Cheng H. Robust reputation-based ranking on bipartite rating networks. In: Proceedings of the 2012 SIAM international conference on data mining. SIAM; 2012. p. 612–623.

[32] Li B, Li RH, King I, Lyu MR, Yu JX. A topic-biased user reputation model in rating systems. Knowledge and Information Systems. 2015;44(3):581–607.

[33] Xu J, Zheng Z, Lyu MR. Web service personalized quality of service prediction via reputation-based matrix factorization. IEEE transactions on reliability. 2016;65(1):28–37. 10.1109/TR.2015.2464075

[34] Zheng Z, Zhang Y, Lyu MR. Distributed qos evaluation for real-world web services. In: 2010 IEEE International Conference on Web Services. IEEE; 2010. p. 83–90.

